# Collaboration in a Partnership for Primary Health Care: A Case Study From Papua New Guinea

**DOI:** 10.9745/GHSP-D-23-00040

**Published:** 2024-02-28

**Authors:** Georgina Dove, Angela Kelly-Hanku, Jethro Usurup, Annmaree O’Keeffe, Geoff Scahill, Adam Craig

**Affiliations:** a University of New South Wales, Sydney, Australia.; b Abt Associates, Brisbane, Australia.; c Papua New Guinea Institute of Medical Research, Goroka, Papua New Guinea.; d Abt Associates, Port Moresby, Papua New Guinea.; e The Lowy Institute, Sydney, Australia.; f University of Queensland Centre for Clinical Research, University of Queensland, Sydney, Australia.

## Abstract

**Introduction:**

In low- and middle-income countries, public-private partnerships (PPPs) are often used to support the delivery of primary health care (PHC). We explore the processes of collaboration in a corporate social responsibility investment in PHC that was delivered through a PPP model in Western Province, Papua New Guinea, in 2009–2018 to strengthen PHC services.

**Methods:**

Qualitative interviews were conducted with stakeholders in the PPP (N=20). Key program documents were also reviewed. Data were coded using a general inductive approach, and Actor-Network Theory (ANT) was used to frame the analysis.

**Results:**

Four dominant themes emerged: (1) interpersonal relationships accelerate collaboration, (2) collaboration requires time, (3) formal governance structures encourage collaboration, and (4) internal change disrupts collaboration. The research provides insight into the role of collaboration for more efficient, effective, and impactful design and implementation of PPP for PHC. It makes suggestions for how ANT may be used when designing PPPs and for the ongoing management of relationships between partners. We found ANT to be a useful framework to conceptualize the complex dynamics between the “actors” within the PPP and to identify opportunities for improvement where structural changes may be made to circumvent issues that may compromise effective collaboration.

**Conclusion:**

Collaboration is key to the success of PPPs for PHC. We found collaboration is not formulaic but is driven by actors, relationships, time, and governance. ANT can assist in designing, understanding, and managing the complex relationships between stakeholders of a PPP, who often come with diverse agendas, experiences, values, and perspectives.

## Abstract (precis)

Four key factors that influence collaboration in a public-private partnership (PPP) are relationships, time, governance, and the impact of change. Incorporating these factors into PPP design and implementation in similar settings can increase coordinated action and improvements in primary health care.

## Teaser

**Key Findings:**

- Collaboration in a public-private partnership (PPP) to strengthen primary health care services was accelerated by strong interpersonal relationships, nurtured through partners’ investment of time and effort, and encouraged by having formal governance structures.
- Changes such as the introduction of new partners and the planned exit of the implementation partner disrupted collaboration in the PPP.
- The Actor-Network Theory can assist PPPs in framing and reviewing collaborative processes and membership and can provide real-time opportunities for improvement and action.

**Key Implications:**

- Organizations participating in PPPs for PHC should engage in thoughtful and considered identification and review of stakeholders and ensure the roles and responsibilities of each stakeholder are understood.
- Organizations implementing PPPs should recognize the factors that support and impede open communication and mutually beneficial collaboration.
- Organizations implementing PPPs should seek national-level policies and guidelines that assist local-level partnerships with forming, operating, and exiting with sustainable outcomes.

## INTRODUCTION

Collaborative action to achieve primary health care (PHC) is a commitment of the Astana Declaration on primary health care.^1^ Public-private partnerships (PPPs) are a model of stakeholder collaboration widely used for infrastructure and service delivery.^2^^,^^3^ In the health sector, PPPs were first documented in the 1990s to support drug and product development, conduct research, develop infrastructure, and provide training.^4^^–^^7^ In low- and middle-income countries where resources for health service delivery are severely limited, PPPs are commonly established to support the delivery of PHC services and extend the reach of government health services,^8^ for example, malaria control in Tanzania,^9^ TB control in Ghana,^10^ and primary health services in Pakistan.^11^ Corporate social responsibility initiatives can be the funding source for PPPs.^12^^–^^14^

The World Bank has designated Papua New Guinea (PNG) as a lower-middle-income country,^15^ with an estimated population of 11.7 million people in 2021.^16^ Eighty percent of PNG’s population live in rural and remote locations,^17^ many in small and extremely isolated villages accessible only by a multiday overland trek. Although it is geographically the largest province in PNG, Western Province, located on the western boundary of the country, has the smallest population (approximately 315,200).^16^ This remote and dispersed population presents challenges for delivering PHC services.

PHC services in PNG are delivered by a range of government, faith-based, nongovernmental, and private providers. Faith-based organizations provided 50% of all rural health services in 2015,^17^ yet there are few published examples of health service organizations working together to deliver services.

Although the literature has examined the use of PPPs to deliver PHC services in low- and middle-income countries,^18^^–^^23^ little attention has been given to exploring the relationships and processes that contribute to collaboration within a PPP for PHC. Collaboration is considered a critical component of the effective governance of a health system.^24^^–^^28^ To address this gap, we used a case study of a corporate social responsibility investment using a PPP mechanism between a mining company, a contracted implementation company, community members, and health service providers in Western Province, PNG, to explore collaboration in a PPP.

## PUBLIC-PRIVATE PARTNERSHIP TO STRENGTHEN PRIMARY HEALTH CARE

The PPP aimed to strengthen existing health services and improve access to health services in the partnership’s catchment areas, which were communities residing within Western Province, PNG, and impacted by the mining operations of Ok Tedi Mining Limited. Administratively, Western Province is divided into 3 districts demarcated by the Fly River: the North Fly District, Middle Fly District, and South Fly District. The capital, Daru, is an island.

The public-private partnership in Western Province aimed to strengthen existing health services and improve access to services in 3 districts.

**FIGURE 1:**
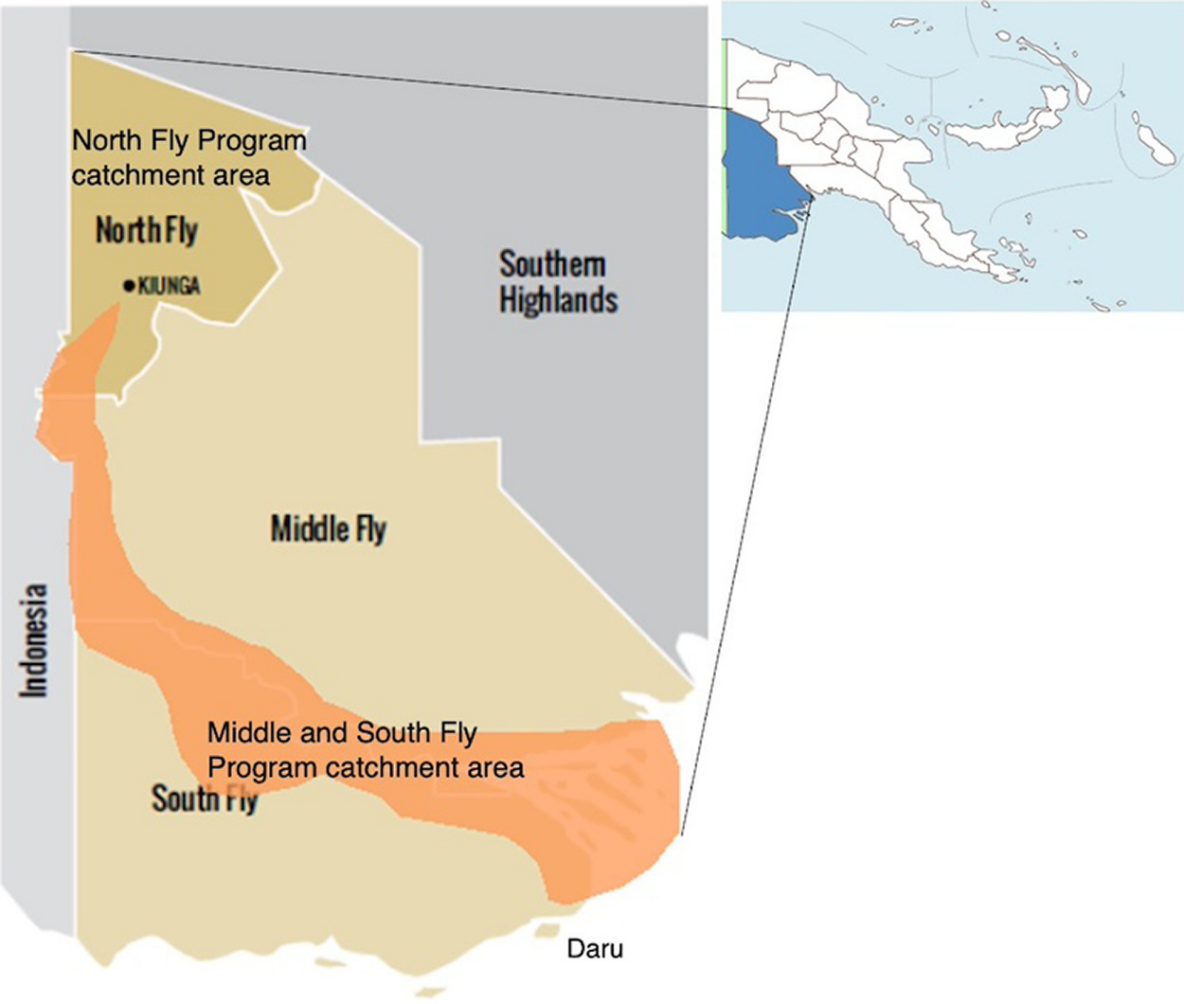
Map of Western Province, Papua New Guinea, Showing Catchment Areas Where the Public-Private Partnership Operated Health Programs

Operationally, the PPP designed and implemented 2 health service development programs: the North Fly Health Services Development Program and the Community Mine Continuation Agreement Middle and South Fly Health Program. The North Fly Health Services Development Program was conceived as a 5-year program (2009–2013) and provided PHC services to the population of the North Fly district (about 108,250 people).^16^ The program was extended to 2018 to align with a second program, the Middle and South Fly Health Program, that operated from 2013 to 2018. PHC services under the Middle and South Fly Program were offered to those who resided in recognized mine-affected communities (about 77,400 people) (Figure 1).^29^

### Partners and Funding

Since 1981, Ok Tedi has operated a large open-cut copper and gold mine in Western Province. Driven by recognition of the poor health status of the surrounding population^30^ and regulatory requirements for mining, in 2008, Ok Tedi initiated and funded the PPP. Over the 10 years of PPP implementation, Ok Tedi provided 101 million PNG Kina (approximately US$28 million) in funding to the partnership.

The other partners of the PPP were the Western Province administrators; district health services; local faith-based health services based within Western Province, including Catholic Church Health Services and Evangelical Church of PNG Health Services; and Abt Associates (known at the time as JTA International), a global development and social impact company. Ok Tedi contracted Abt Associates to manage the PPP from 2009 to 2018.

Before the PPP’s establishment, the partners had no formal working relationship. One of Abt Associates’ first tasks was to bring the partners together to codesign the PPP and develop a nonbinding partnership charter. This charter established the roles and responsibilities of all the partners, a statement of shared philosophy, and the PPP’s governance and management structures. The charter guided daily cooperation in the early stages of the PPP when working relationships were still forming. Throughout the PPP, partners shared their targets and plans for health service delivery and identified gaps and challenges in service delivery and possible solutions. These activities worked toward providing services in all communities while reducing duplications. Table 1 summarizes each partner’s responsibilities.

**TABLE 1.** Partner Responsibilities in the Public-Private Partnership to Improve Primary Health Care, Western Province, Papua New Guinea

**Partner Organization**	**Primary Responsibilities**
Ok Tedi Mining Limited	Funding Overall strategic direction of program
Abt Associates	Manage the daily program implementation Convene partnership meetings Communicate and collaborate with other partners Identify areas of need within catchment services and populations and engage in collaborative decision-making about potential responses Employ health workers to work with local health services Report on partnership funding and achievements
Western Province administration (government)	Provide strategic direction for the province through annual plans and other strategies and plans Actively participate in partnership activities and governance Communicate and collaborate with other partners Identify areas of need within catchment services and populations and engage in collaborative decision-making about potential responses
District health services (government)	Actively participate in partnership activities and governance Communicate and collaborate with other partners Identify areas of need within catchment services and populations and engage in collaborative decision-making about potential responses
Local faith-based health services	

### Health Services Provided

PHC services were provided to community members within the program and providers’ catchment areas. All health service providers in the program catchment area were partners in the PPP, and health services in each community were offered by 1 service provider. The partnership’s aim to strengthen existing services aligned with the goals outlined in the National Health Plan 2011–2020^31^ and the National Health Service Standards.^32^ Activities under the PPP included health promotion, disease prevention, and treatment in maternal and child health, as well as communicable disease control for malaria, TB, and HIV. The partnership also contributed to strengthening “enablers” of health care (i.e., infrastructure, transport, health worker education, and medical supply chain).

### Monitoring and Evaluation

At the national level in PNG, health data are collected and reported through the National Health Information System, enabling monitoring of health indicators at the district and provincial levels. The PPP submitted all service delivery information as required and also sought to strengthen health information management in existing health services through training in quality, timely, and complete reporting and encouraging partner organizations to use data for real-time decision-making.

**FIGURE 2:**
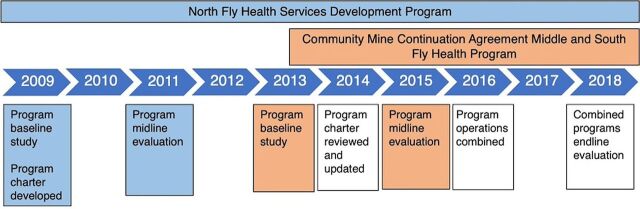
Timeline of Implementation and Review of the Primary Health Care Partnership Programs in Western Province, Papua New Guinea

The national health indicators were incorporated into the partnership’s monitoring and evaluation framework. The indicators and the partnership approach used in the programs were reviewed throughout implementation (Figure 2). These evaluations were disseminated via Abt Associates and Ok Tedi verbally and in print to all partners, communities in Western Province, government agencies, and other interested parties, as well as made publicly available on Ok Tedi and Abt Associates’ websites. While there were no formal reporting or implementation requirements of the partnership from the PNG Government, Abt Associates made every endeavor to share partnership information and progress with the national government.

### Achievements and Challenges

The partnership contributed to improvements in health service performance. Western Province was ranked the most improved performer of all 22 provinces in the 2017 Sector Performance Annual Review.^29^ Further examples include an increase in the number of outreach clinics in North Fly District per 1,000 children aged younger than 5 years from 22 in 2009 to 37.5 in 2017,^29^ and an increase in measles immunization coverage in Middle and South Fly Districts for children aged younger than 1 year from 51% in 2013 to 62% in 2017.^29^

The partnership faced several external and internal challenges. External and practical challenges included geography and extreme weather events, as well as limited transport, human resources, and medical supplies^29^ that impeded service delivery. Significant internal challenges included working with government services to transition program activities to their stewardship and fostering sustainability.^29^ The partnership had a transition plan that it actively worked toward; however, the prospect of no additional partnership funding raised concerns for all partners that the activities would not be sustained at the same level as they had been in the partnership.

## METHODS

### Data Collection

Data were collected during the combined programs’ endline evaluation in 2018 using 2 methods. First, key informant interviews were conducted with a sample of stakeholders of the PPP (N=20). Interviewees were purposefully selected to ensure that the views of stakeholders from different organizations with different roles and of different genders were collected. Interviewees were invited to participate by a representative of Abt Associates in PNG who was not involved in the daily operations of the PPP. Most interviews were conducted individually (2 included 2 participants). All interviews were semistructured in nature and used a guide (Supplement) to allow participant-derived content to be explored while covering core content areas. The interviews collected data about interviewees’ demographics and roles within the PPP; interviewees’ experience and views of the PPP, with a focus on partnership forming and governance dynamics; and views on factors that enabled or eroded program achievements, partnership stability, and PPP sustainability. All interviews were conducted by an evaluation consultant contracted to Abt Associates in English and at the interviewees’ usual place of employment. Interviews lasted an average of 36 minutes and were recorded and transcribed verbatim. Informed written consent was received from all participants.

Second, relevant PPP-related documents, including activity plans, correspondence, meeting records, and monitoring and evaluation reports, were reviewed, and data related to the governance structures, engagement, and conflict (and conflict resolution) were extracted. These data were recorded verbatim in Microsoft Excel.

### Data Analysis

The collected data were coded using NVivo 12 and, drawing on the work of Braun and Clarke^33^ and Terry et al.,^34^ thematically analyzed by the lead author using a generalized inductive approach. Results were grouped by components of Actor-Network Theory (ANT) to support analysis.

### Dissemination of Results

The combined programs’ endline evaluation results, including a summary of the key informant interviews that inform this article, were disseminated as outlined under the monitoring and evaluation section. All partner organizations provided letters of support for the interviews to be used in this study, and the letters were included in the ethics applications. Partner organizations and interviewees provided written informed consent for the interviews to be analyzed and used in publications.

### Theoretical Framework

We adopted the ANT as a conceptual approach to frame the exploration of the relationships and processes that contributed to the collaboration and governance of the PPP. Conceived by Callon^35^ and redefined by Latour,^35^ ANT provides a framework for understanding how human and nonhuman “actors” or entities interact within a network, defined as “a set of human and nonhuman entities connected within a network.”^35^ Examples of nonhuman entities are animals, written documents, and technology.^35^

ANT has been used to describe and explain intersectoral collaborations that aim “to reduce social inequalities in health.”^35^ Law^36^^–^^38^ believed the “actor-network theory is all about power,” which is pertinent to partnerships and their collaborative processes and governance.

ANT is concerned with defining who (or what) the actor/s are, how they interact with each other, and what influence the actors and their interactions have on a network. This principle is known as translation.^35^ ANT considers 5 concepts of translation, and these are defined at relevant points in the discussion section of the article. For this case study, the network was the PPP, and we focused our analysis on the human actors and their interaction by looking at the 5 concepts or phases within translation. Although other models of collaboration could also be applied to this case study, ANT was deemed a useful analytical framework for this study, as it explores the processes of engagement and the impact relationships have on a network or, in this case, partnership outcomes.

### Ethical Approval

Ethics approval for the research study was granted by the Papua New Guinea Institute of Medical Research (IMR 1717), Papua New Guinea National Department of Health (MRAC 17.49), and the University of New South Wales Sydney’s Human Research Ethics Committee (HC 180124).

## RESULTS

Twenty key informant interviews were conducted, including 3 with senior government administration staff, 2 with government health services staff, 5 with faith-based health services staff, 4 with representatives of Ok Tedi, and 6 with program staff of Abt Associates (Table 2). The sex of participants was collected but has not been reported alongside quotes to ensure anonymity.

**TABLE 2.** Characteristics of Key Informant Interview Participants

**Characteristic**	**No. (%)(N=20)**
Sex	
Female	6 (30)
Male	14 (70)
Nationality	
Papua New Guinean	16 (80)
Non-Papua New Guinean	4 (20)
Employee/organization affiliation	
Provincial government administration	3 (15)
Government health service	2 (10)
Faith-based health service	5 (25)
Ok Tedi Mining Limited	4 (20)
Abt Associates	6 (30)

Four dominant themes relating to collaboration in a PPP for PHC in PNG were derived from the inductive thematic coding process. We discuss these in the following sections.

### Interpersonal Relationships Accelerate Collaboration

We found that, during the establishment of the partnership, the implementation partner emphasized and invested time and effort in developing interpersonal relationships between partnership members and that this investment was unilaterally seen by stakeholders as fundamental to success. We found 3 key considerations that fostered interpersonal relationship building: understanding other partners’ perspectives, communication, and personality attributes.

Stakeholders unilaterally perceived that the implementation partner’s investment of time and effort in developing interpersonal relationship between partners as fundamental to success.

First, building relationships required the partners within the PPP to invest in and take time to develop an understanding of the other partners’ perspectives, motivations, and ways of engaging and operating, not only within the PPP but also more broadly within the existing health sector landscape.

The value of interpersonal relationships to the partnership was expressed clearly by 1 interviewee.

*…if I have this good personal relationship with you [a counterpart from within the PPP], it’s a little bit easier for us to talk…* —Abt Associates representative

Several interviewees commented that not everything went smoothly in establishing relationships. For example, interviewees noted that the North Fly Service Development Program (the first program within the partnership) encountered several relationship-related challenges when first established. Interviewees said these challenges stemmed from a lack of shared understanding among the partners about the purpose of the PPP, individuals’ roles, and agencies’ relationships with each other. Interviewees reported that the lack of common understanding at the outset of the North Fly Health Program led to difficulties engaging with partner organizations and communities. These difficulties were related to Ok Tedi’s involvement, as partners and communities saw program staff as being aligned with Ok Tedi, and there was suspicion about implications for communities if they did not engage with the program. To address this perception, the uniform worn by program staff was changed and the high-visibility markers that identified the uniform as being associated with a mine were removed.

Second, communication was a commonly reported factor that enabled partnerships to form. Interviewees overwhelmingly identified that successful actions in developing interpersonal relationships were those based on respectful and culturally appropriate communication—in particular, communication that was at a personal and not just professional level and was in person. The use of in-person meetings was important to making decisions about resources and priorities and played a key role in relationship building.

Interviewees expressed that the absence of in-person communication contributed to the distance between organizations’ offices and limited interaction.

*It’s a little bit hard to collaborate with our partners because the program operates out of Kiunga. The provincial head office sits in Daru. Sometimes, most times, it has a bearing on how we try to interact with the health sector leadership.* —Abt Associates representative

Third, while the relationships between all actors were important, interviewees noted that some actors had inherent personality attributes that led them to play particularly important roles and be considered “key drivers of the partnership.” The unique skills and attributes identified were in building and maintaining relationships, including having influence with partner organizations and the ability to bring them together; being resilient and not giving up when faced with resistance; and modeling a commitment to working for the partnership in the long term and to achieving the program aims. One interviewee at Ok Tedi summed up these attributes by saying, “I would go so far as to say [program manager] is exceptional.”

In all interviews, we noted the use of inclusive language and possessive pronouns, such as “us,” “our,” and “we,” when discussing the program and partnership.

### Collaboration Requires Time

Our research found that the temporality of the partnership was identified as central to collaboration in 2 ways. First, time was required to build relationships and trust and establish new and mutually beneficial ways of working. Second, time was required to see tangible results from the collaboration.

Interviewees reported that collaboration between partners improved over time as relationships were established and strengthened. They reported initial skepticism about the partnership, Abt Associates’ role in the program, and partners’ unwillingness to engage with the program at its commencement.

Over time, relationships in the partnership were impacted by changes in membership and by each organization’s degree of engagement in the partnership. Membership over its 10-year history was not static, although all original member organizations (district and faith-based health service organizations) remained throughout. At an organizational level, interviewees indicated that at the time of its inception in 2009, almost all health service organizations operating in the program catchment area were represented in the partnership. An Ok Tedi interviewee described this as “a fair distribution on the partners.” When new actors were identified and invited to join the partnership, effort was required to form new and maintain existing relationships.

By the end of the program, interviewees across different organizations considered the collaboration to be “very strong,” with organizations seen to complement each other in their activities. Examples provided by interviewees to support this claim included 28 organizations represented at a stakeholder meeting in 2018, the speed with which organizations met and acted in the event of an emergency, and that the program was no longer directly involved in the operational activities of all partners.

The second time-related factor was that evidence of tangible results from the partnership also took time to appear. The provision of material assistance, such as radio installations and transport assets, was viewed as a positive contribution of the PPP because it improved health workers’ ability to deliver PHC services. Interviewees believed that material assistance had a positive influence on the initial development of relationships. In turn, the strengthening of relationships led to greater collaboration in service delivery and improvements in health status indicators, as reported by 1 interviewee.

*We say indicators for North Fly in those days were really up high because we were really working in partnership and we brought services to people.* —Faith-based health service representative

One interviewee stated that their organization, a later inclusion in the partnership, was able to leverage the partnerships built over time by the program and used that to its advantage in operating in the province.

Interviewees shared examples of coordinated and collaborative work undertaken in the case study, including combining resources to conduct joint health outreach patrols.

### Formal Governance Structures Encourage Collaboration

One objective of the programs was to strengthen partnerships and coordination,^19^ and all interviewees stated that the governance structures established from its commencement in 2009 facilitated this. Interviewees reported that the governance arrangements were recorded in a program charter that defined stakeholders’ roles and responsibilities and contained a “statement of shared philosophy” that was agreed upon by all parties. In 2014, the charter was reviewed at the commencement of the second program and updated to include new partners; no other substantial changes were made.

Interviewees stated that the governance structures established from its commencement in 2009 facilitated strong partnerships and collaboration.

Interviewees agreed that another governance structure that facilitated collaboration was the program activity groups. Health workers from all partner organizations were represented in these issue-specific groups, which were convened regularly to plan and conduct collaborative activities. Interviewees reported that the groups for maternal and child health, TB, and scholarships were a coordination mechanism that worked well in North Fly due to regular meetings where all organizations were represented and the fact that groups had detailed schedules of activities. In particular, the Maternal and Child Health Program Activity Group was noted as effective in reaching remote communities, and the Tuberculosis Program Activity Group was exemplified to communicate and coordinate with all partners.

### Internal Change Disrupts Collaboration

Our research found a strong sense from interviewees that, after some initial challenges, collaboration between the actors was very strong in the first 5 years of the partnership when there was only 1 program in operation. Interviewees identified 2 major internal changes in the partnership that disrupted this collaboration.

The first disruption was the introduction of new partners through the addition of a second program within the partnership in 2013 in the Middle and South Fly districts. The second program commenced 5 years after the first program, and soon after, the 2 programs were operated as a single program. Despite building on the existing relationships from the first program, the consensus from interviewees who were involved in both programs was that the partnership was strongest in those first 5 years.

The second disruption or destabilizing factor in the PPP was the planned exit of the implementation partner, Abt Associates, upon completion of its contract. The partnership itself was anticipated to continue, and meeting minutes and program reviews show that efforts were made from the commencement of the program to ensure that roles and responsibilities held by Abt Associates were redistributed to other actors. Interviewees acknowledged that the program initiated a transition plan and a series of milestones for handing over program activities to partners. No interviewees reflected on whether the transition plan had an impact on collaboration, either positive or negative.

Interviewees stated that Abt Associates was clear in its intention to transfer responsibility for the activities to partner organizations starting in 2015. One interviewee reflected that local health service organizations did not accept the responsibility, while another echoed and expanded on that.

*They’ve [the program] been trying to hand it over, it’s just that there is resistance on the ground.* —Faith-based health service representative

In contrast, another perspective was that the implementation partner could have been more definitive in upholding the transition plan by relinquishing its various responsibilities at the specified time to force partners to take over.

Our research found a desire among partners to retain strong coordination and collaboration once the implementation partner had exited the partnership. However, there were mixed responses from interviewees as to whether they believed the partnership structure would be sustained without the implementation partner.

All interviewees felt very strongly that stakeholders came together and collaborated because of and with the assistance of the coordination provided by the implementation partner Abt Associates. Some indicated that once the program ended and Abt Associates exited, the coordination of and communication between partners would not continue. Other interviewees stated that the existence of the Western Province Health Steering Committee before the commencement of the program was an indication that the meetings and coordination would continue after Abt Associates exited.

Interviewees reported that the planned exit of Abt Associates would impact the sustainability of the partnership. They expressed doubt about the continuity of stakeholders coordinating their activities if the funding provided through Abt Associates to bring all stakeholders together to meet in person regularly was no longer available. Interviewees identified that it was the government’s (provincial and district services) responsibility to coordinate and proactively bring stakeholders together; however, provincial and district administrations stated they did not have funding available to bring stakeholders together.

## DISCUSSION

We thematically analyzed data collected from documents and interviews with 20 stakeholders of a PPP for PHC in Western Province, PNG, to explore the relationships and processes that contribute to successful collaboration in a PPP. We identified 4 key themes: the impacts of interpersonal relationships, temporality, formal governance structures, and disruptions to collaboration. We then used ANT to explore how PPP actor roles and interactions influenced partnership collaboration.

ANT places emphasis on identifying actors and defining their roles and responsibilities as 2 key steps in developing and managing relationships. In the case study, the identification of actors was undertaken carefully at the initiation of the partnership and was an ongoing activity throughout the program duration. As new health service delivery organizations were established in the program catchment location, they were identified and invited to join the partnership. Identified actors and their roles and responsibilities were documented in the program charter, which was updated once in 2014.

The relationships between organizations and individual actors were found to be critical to the progression of the partnership, and we found that relationships were considered stronger over time. The alignment of actors’ interests and the acceptance of their roles and responsibilities was strongest in the first 5 years of implementation and appeared to weaken temporarily when new stakeholders were introduced, having a destabilizing effect on the partnership. This alignment had strengthened again by the end of the partnership. Attention to building, maintaining, and reviewing relationships should be incorporated into all stages of a PPP.

The alignment of actors’ interests and the acceptance of their roles and responsibilities was strongest in the first 5 years of implementation and appeared to weaken temporarily when new stakeholders were introduced, having a destabilizing effect on the partnership.

We found that collaborative action, including coordination and communication, improved over time. The inclusive language used in the interviews to refer to the partnership by a range of actors, not just by Abt Associates representatives, suggests that, at least subconsciously, there was an important degree of ownership and, as such, aligned interests and strong relationships. In designing a PPP, the value and necessity of time should not be underestimated and should be incorporated into proposed partnership processes and expected outcomes.

ANT encourages practitioners to consider the stability of networks as a component of function. Although network development is not considered linear in ANT,^35^^,^^38^ a stabilized network has stronger connections and alignment between actors, which brings greater cohesion and efficiency within the network and reduced individual identity and conflict.^35^ The case study demonstrates different levels of stability evident throughout the programs’ 10-year duration as actors, actions, and alignment varied. This was a useful reflection as it allowed us to identify the impact of internal and external variations. ANT offers a framework for identifying and addressing fluctuations in stability that may occur in health partnerships in other low- and middle-income countries.

The research found the PPP partners valued the coordination function provided by the implementation partner but were concerned that, at the completion of the implementation partner’s involvement, collaboration would cease. This point was highlighted by interviewees who expressed that partners were unwilling to take on the coordination responsibilities. To lessen the effects of program closure, transition planning that commences well in advance and incorporates the external resources required to continue collaboration is prudent. Advanced transition planning is of particular importance in low-resource settings such as low- and middle-income countries. Policy and guidelines that support PPP design and build the scaffolds needed for sustainability would be of use. A national policy could be developed collaboratively with input from the PNG government, health service, and industry organizations with prior experience in PPP implementation, corporate social responsibility, and peak bodies.

ANT provides a means to understand the processes of PHC partnerships between multiple actors or stakeholders and can be used at various stages of partnership formation. Ideally, the concepts would be considered and addressed upon establishment of a partnership but may be just as relevant during and after establishment (i.e., during implementation) to review progress and processes and to adapt and improve where required. This case study review is a retrospective analysis of the partnership. An earlier analysis, during program implementation, could have benefited the stabilization and sustainability of the partnership. A framework and time frame for regular partnership reviews, aligned with ANT concepts, should be considered on commencement of a partnership program.

In reflecting on the central tenet of ANT, translation, we believe the case study partnership addressed all stages and concepts within translation to varying degrees. This variability is inherent in complex programs. Thus, the ANT concepts provide guidance for successful collaboration in PHC partnerships in low- and middle-income settings.

Differences in ethnicity and language present opportunities for bias and power imbalances. While most program staff and partner representatives were Papua New Guinean, the interviews and data analysis for this study (as a component of the program endline evaluation) were led by 2 different authors, both Australian. This was a consideration for conducting the interviews in English, although English was also the main language used in the operation of the PPP. The consultant provided objectivity; however, we acknowledge that interviewees may have been reserved in their responses because of the consultant’s engagement by Abt Associates.

### Limitations

Our study had limitations. The PPP was implemented in a complex and dynamic setting, and it is therefore possible that nuances have been missed. Many of the interviewees were also beneficiaries of the PPP and may, therefore, hold (and have reported) views that were biased. Data were collected in 2018, near the end of the 10-year program, and interviewees’ insights may, therefore, be subject to recall bias. Although they were not responsible for data collection, GD, GS, and JU are employees of Abt Associates and were part of the PPP implementation team, which may have biased analysis. AO was contracted by Abt Associates to conduct the interviews but was not otherwise involved in the PPP implementation; all other authors had no connection to the PPP and Abt Associates.

Ethnicity and language may also have led to biases in data collection and analysis as JU was the only author from Papua New Guinea, and the interviews were conducted in English rather than Tok Pisin. Despite these limitations, our research is valuable in that it is the first to apply ANT to a PPP and provides novel insights into the processes that enable and inhibit collaboration in PPP for PHC in the PNG context. Although the learning from this case study is specific to PNG and the program, it will be of interest to others establishing and implementing partnerships to improve PHC.

## CONCLUSION

Partnership collaboration for PHC is not formulaic; rather, it is the result of its purpose, actors, and relationships. Our research found collaborations can be fostered by supporting the development of interpersonal relationships, allowing time for partners to build trust and establish working relationships, establishing clear and mutually valued governance structures, and managing disruptive change proactively. We found that ANT provides a useful framework through which actors in a PPP and their relationships can be understood, analyzed, and supported and provides a framework for designing collaboration in a PPP.

## Supplementary Material

GHSP-D-23-00040-supplement.pdf

## Notes

**Peer Reviewed**

**First published online:** January 22, 2024.
