# Supplementary material for: Collaboration in a Partnership for Primary Health Care: A Case Study From Papua New Guinea
Source: Glob Health Sci Pract. 2024 Feb 28;12(1):e2300040. doi: 10.9745/GHSP-D-23-00040 (PMC10906555; doi:10.9745/GHSP-D-23-00040)
Supplement: GHSP-D-23-00040-supplement.pdf [file GHSP-D-23-00040-supplement.pdf]

Supplement to: Dove G, Kelly-Hanku A, Usurup J, O'Keeffe, Scahill G, Craig A. Collaboration in a partnership for primary health care: a case study from Papua New Guinea. *Glob Health Sci Pract*. 2024;12(1):e2300040. https://doi.org/10.9745/GHSP-D-23-00040

SUPPLEMENT

Interview Guide

1. Program Partnership
   1.1. Please tell me about your experience of being part of the Partnership?
   - Are you satisfied with how the Partnership has operated?

   1.2. How are issues raised and addressed through the Partnerships?
   - Can you give an example of an issues raised and the action taken?

   1.3. Reflecting on your involvement over the life of the Program, have there been changes in the way the partnership has operated?
   - Can you give an example of how the partnership has changed?

2. Program progress
   2.1. What were your expectations of the Program?
   - Has the Program met your expectations?
   - In what way had the Program met/not met your expectations?

   2.2. What do you think the Program has achieved?
   - Have you noticed any changes in the Program catchment area?

3. Sustainability
   3.1. What do you think needs to happen to ensure the outcomes of the Program are maintained after completion in 2018?
   - What additional support does your organization need?

   3.2. What do you think needs to occur for health services in the Program area to be sustainable after the Program ends?
